# Ethyl 2-[4-(1,3-benzothiazol-2-yl)­anilino]acetate

**DOI:** 10.1107/S1600536810029442

**Published:** 2010-07-31

**Authors:** Yong Zhang, Yuan Qu, Bi-lin Zhao

**Affiliations:** a School of Chemical and Materials Engineering, Huangshi Institute of Technology, Huangshi 435003, People’s Republic of China

## Abstract

In the title compound, C_17_H_16_N_2_O_2_S, the dihedral angle between the benzothia­zole ring system and the benzene ring is 1.20 (2)°. The substituted amino substituent is in an extended conformation with an N—C—C—O torsion angle of 179.4 (3)°. In the crystal structure, pairs of mol­ecules are connected by inter­molecular N—H⋯O and weak C—H⋯O hydrogen bonds, forming centrosymmetric dimers.

## Related literature

For background to thio­flavin T (ThT), a benzothia­zole dye that exhibits enhanced fluorescence upon binding to amyloid fibrils, and its derivatives, see: Kung *et al.* (2001 ▶); Qu *et al.* (2007 ▶); Zhang & Zhao (2009 ▶). For the synthesis, see: Stephenson *et al.* (2007 ▶).
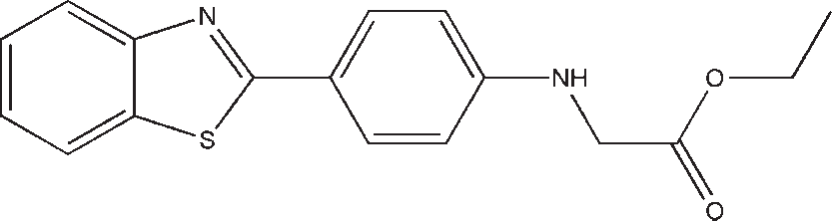


## Experimental

### 

#### Crystal data

C_17_H_16_N_2_O_2_S
*M* *_r_* = 312.38
Monoclinic, 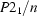

*a* = 5.6303 (1) Å
*b* = 26.1604 (5) Å
*c* = 10.5989 (2) Å
β = 98.294 (1)°
*V* = 1544.79 (5) Å^3^
*Z* = 4
Mo *K*α radiation
μ = 0.22 mm^−1^
*T* = 298 K
0.36 × 0.24 × 0.21 mm

#### Data collection

Bruker SMART CCD area-detector diffractometer
Absorption correction: multi-scan (*SADABS*; Sheldrick, 1996 ▶) *T*_min_ = 0.926, *T*_max_ = 0.956
11631 measured reflections
3808 independent reflections
3015 reflections with *I* > 2σ(*I*)
*R*_int_ = 0.076

#### Refinement

*R*[*F* ^2^ > 2σ(*F* ^2^)] = 0.058
*wR*(*F* ^2^) = 0.145
*S* = 1.07
3808 reflections
203 parameters
1 restraint
H atoms treated by a mixture of independent and constrained refinement
Δρ_max_ = 0.39 e Å^−3^
Δρ_min_ = −0.30 e Å^−3^

### 

Data collection: *SMART* (Bruker, 2007 ▶); cell refinement: *SAINT-Plus* (Bruker, 2007 ▶); data reduction: *SAINT-Plus*; program(s) used to solve structure: *SHELXS97* (Sheldrick, 2008 ▶); program(s) used to refine structure: *SHELXL97* (Sheldrick, 2008 ▶); molecular graphics: *PLATON* (Spek, 2009 ▶); software used to prepare material for publication: *SHELXTL* (Sheldrick, 2008 ▶).

## Supplementary Material

Crystal structure: contains datablocks global, I. DOI: 10.1107/S1600536810029442/lh5087sup1.cif

Structure factors: contains datablocks I. DOI: 10.1107/S1600536810029442/lh5087Isup2.hkl

Additional supplementary materials:  crystallographic information; 3D view; checkCIF report

## Figures and Tables

**Table 1:** Hydrogen-bond geometry (Å, °)

*D*—H⋯*A*	*D*—H	H⋯*A*	*D*⋯*A*	*D*—H⋯*A*
C12—H12⋯O2^i^	0.93	2.60	3.390 (2)	144
N2—H2*A*⋯O2^i^	0.85 (1)	2.40 (1)	3.188 (2)	154 (2)

Symmetry code: (i) 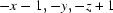.

**Fig. 1.**
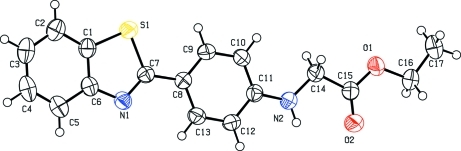
The molecular structure of (I), with displacement ellipsoids drawn at the 50% probability level.

**Fig. 2.**
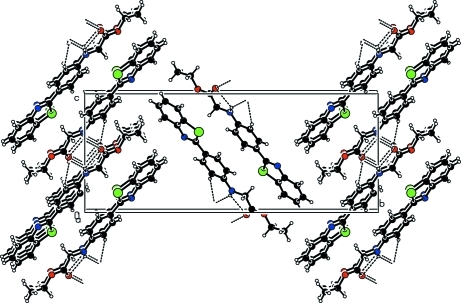
Part of the crystal structure of (I) showing hydrogen bonds as dashed lines.

## supplementary crystallographic information

### Comment

Thioflavin T (ThT) is a benzothiazole dye that exhibits enhanced fluorescence upon binding to amyloid fibrils and is commonly used to diagnose amyloid fibrils, both ex vivo and *in vitro*. Many derivatives of thioflavin T have been synthesized and evaluated recently (Kung *et al.*, 2001; Qu *et al.*, 2007; Zhang, *et al.*, 2009). We are interested in developing fluorescent probes that are expected to bind to hydrophobic sites in proteins. With this in mind, the title compound, (I), was synthesized and we reported the crystal structure herein.

In the molecular structure (Fig. 1), the dihedral angle between the benzothiazole ring system and the benzene ring is 1.20 (2)°. The substituted amino substituent is in an extended conformation with an  N—C—C—O torsion angle of 179.4 (3)°. In the crystal structure, pairs of molecules are connected by intermolecular N—H···O and weak C-H···O hydrogen bonds to form centrosymmetric dimers (Fig. 2).

### Experimental

Compound (I) was synthesized according to the method described by Stephenson *et al.* (2007). Pale yellow single crystals suitable for an X-ray diffraction study were obtained by slow evaporation of an methanol solution.

### Refinement

All H atoms were placed in idealized positions [*C*—*H*(methyl)=0.96 Å, 0.97Å (methylene) and 0.93 Å (aromatic),with *U*_iso_(H)= 1.5*U*_eq_(methyl C) 1.2*U*_eq_(other C). N-bounded hydrogen atom was found from the difference map and refined with the restraint of N—H = 0.86 (1)Å and *U*_iso_(H) = 1.2 *U*_eq_(N).

### Crystal data

**Table:**

C_17_H_16_N_2_O_2_S	*F*(000) = 656
*M**_r_* = 312.38	*D*_x_ = 1.343 Mg m^−^^3^
Monoclinic, *P*2_1_/*n*	Mo *K*α radiation, λ = 0.71073 Å
Hall symbol: -P 2yn	Cell parameters from 3368 reflections
*a* = 5.6303 (1) Å	θ = 2.5–26.1°
*b* = 26.1604 (5) Å	µ = 0.22 mm^−^^1^
*c* = 10.5989 (2) Å	*T* = 298 K
β = 98.294 (1)°	Block, pale-yellow
*V* = 1544.79 (5) Å^3^	0.36 × 0.24 × 0.21 mm
*Z* = 4	

### Data collection

**Table:**

Bruker SMART CCD area-detector diffractometer	3808 independent reflections
Radiation source: fine-focus sealed tube	3015 reflections with *I* > 2σ(*I*)
graphite	*R*_int_ = 0.076
φ and ω scans	θ_max_ = 28.3°, θ_min_ = 2.1°
Absorption correction: multi-scan (*SADABS*; Sheldrick, 1996)	*h* = −7→6
*T*_min_ = 0.926, *T*_max_ = 0.956	*k* = −34→34
11631 measured reflections	*l* = −10→14

### Refinement

**Table:**

Refinement on *F*^2^	Primary atom site location: structure-invariant direct methods
Least-squares matrix: full	Secondary atom site location: difference Fourier map
*R*[*F*^2^ > 2σ(*F*^2^)] = 0.058	Hydrogen site location: inferred from neighbouring sites
*wR*(*F*^2^) = 0.145	H atoms treated by a mixture of independent and constrained refinement
*S* = 1.07	*w* = 1/[σ^2^(*F*_o_^2^) + (0.0584*P*)^2^ + 0.2785*P*] where *P* = (*F*_o_^2^ + 2*F*_c_^2^)/3
3808 reflections	(Δ/σ)_max_ < 0.001
203 parameters	Δρ_max_ = 0.39 e Å^−^^3^
1 restraint	Δρ_min_ = −0.30 e Å^−^^3^

### Special details

**Table:**

Geometry. All e.s.d.'s (except the e.s.d. in the dihedral angle between two l.s. planes) are estimated using the full covariance matrix. The cell e.s.d.'s are taken into account individually in the estimation of e.s.d.'s in distances, angles and torsion angles; correlations between e.s.d.'s in cell parameters are only used when they are defined by crystal symmetry. An approximate (isotropic) treatment of cell e.s.d.'s is used for estimating e.s.d.'s involving l.s. planes.
Refinement. Refinement of *F*^2^ against ALL reflections. The weighted *R*-factor *wR* and goodness of fit *S* are based on *F*^2^, conventional *R*-factors *R* are based on *F*, with *F* set to zero for negative *F*^2^. The threshold expression of *F*^2^ > σ(*F*^2^) is used only for calculating *R*-factors(gt) *etc*. and is not relevant to the choice of reflections for refinement. *R*-factors based on *F*^2^ are statistically about twice as large as those based on *F*, and *R*- factors based on ALL data will be even larger.

### Fractional atomic coordinates and isotropic or equivalent isotropic displacement parameters (Å^2^)

**Table:**

	*x*	*y*	*z*	*U*_iso_*/*U*_eq_	
C1	0.4095 (4)	0.16282 (7)	1.27189 (19)	0.0459 (4)	
C2	0.5567 (4)	0.18305 (9)	1.3771 (2)	0.0584 (6)	
H2	0.7050	0.1685	1.4067	0.070*	
C3	0.4757 (5)	0.22514 (9)	1.4357 (2)	0.0624 (6)	
H3	0.5712	0.2397	1.5054	0.075*	
C4	0.2521 (5)	0.24629 (8)	1.3919 (2)	0.0614 (6)	
H4	0.2007	0.2748	1.4330	0.074*	
C5	0.1066 (4)	0.22608 (8)	1.2896 (2)	0.0552 (5)	
H5	−0.0426	0.2406	1.2618	0.066*	
C6	0.1840 (3)	0.18348 (7)	1.22724 (19)	0.0429 (4)	
C7	0.1778 (3)	0.12057 (7)	1.08723 (18)	0.0388 (4)	
C8	0.0933 (3)	0.08695 (7)	0.98019 (17)	0.0383 (4)	
C9	0.2312 (3)	0.04687 (7)	0.94479 (19)	0.0459 (5)	
H9	0.3815	0.0408	0.9915	0.055*	
C10	0.1514 (3)	0.01576 (7)	0.84224 (19)	0.0461 (5)	
H10	0.2472	−0.0110	0.8214	0.055*	
C11	−0.0722 (3)	0.02423 (7)	0.76967 (18)	0.0404 (4)	
C12	−0.2144 (3)	0.06424 (7)	0.80651 (19)	0.0444 (4)	
H12	−0.3657	0.0701	0.7608	0.053*	
C13	−0.1326 (3)	0.09459 (7)	0.90894 (19)	0.0440 (4)	
H13	−0.2295	0.1209	0.9315	0.053*	
C14	−0.0362 (3)	−0.04854 (7)	0.62537 (19)	0.0448 (4)	
H14A	−0.0175	−0.0734	0.6941	0.054*	
H14B	0.1222	−0.0394	0.6068	0.054*	
C15	−0.1818 (3)	−0.07146 (7)	0.50852 (19)	0.0439 (4)	
C16	−0.1958 (4)	−0.13779 (8)	0.3566 (2)	0.0503 (5)	
H16A	−0.2157	−0.1138	0.2860	0.060*	
H16B	−0.3530	−0.1502	0.3694	0.060*	
C17	−0.0407 (4)	−0.18121 (8)	0.3288 (2)	0.0621 (6)	
H17A	0.1178	−0.1688	0.3228	0.093*	
H17B	−0.1070	−0.1968	0.2495	0.093*	
H17C	−0.0329	−0.2060	0.3961	0.093*	
N1	0.0567 (3)	0.15892 (6)	1.12304 (15)	0.0441 (4)	
N2	−0.1552 (3)	−0.00386 (7)	0.66362 (18)	0.0538 (5)	
H2A	−0.294 (2)	0.0014 (8)	0.622 (2)	0.065*	
O1	−0.0780 (3)	−0.11295 (5)	0.47181 (14)	0.0506 (4)	
O2	−0.3671 (3)	−0.05410 (6)	0.45639 (16)	0.0655 (5)	
S1	0.46125 (9)	0.11103 (2)	1.17793 (6)	0.05496 (19)	

### Atomic displacement parameters (Å^2^)

**Table:**

	*U*^11^	*U*^22^	*U*^33^	*U*^12^	*U*^13^	*U*^23^
C1	0.0511 (11)	0.0454 (10)	0.0419 (11)	−0.0041 (8)	0.0096 (9)	−0.0032 (8)
C2	0.0616 (13)	0.0634 (13)	0.0489 (13)	−0.0079 (10)	0.0030 (10)	−0.0080 (10)
C3	0.0821 (16)	0.0581 (13)	0.0474 (13)	−0.0199 (12)	0.0104 (11)	−0.0098 (10)
C4	0.0927 (17)	0.0426 (11)	0.0531 (13)	−0.0085 (11)	0.0250 (13)	−0.0090 (10)
C5	0.0695 (13)	0.0426 (11)	0.0554 (13)	0.0028 (9)	0.0160 (11)	−0.0009 (10)
C6	0.0519 (11)	0.0375 (9)	0.0409 (10)	−0.0028 (8)	0.0121 (8)	0.0019 (8)
C7	0.0396 (9)	0.0392 (9)	0.0374 (10)	0.0001 (7)	0.0049 (7)	0.0034 (7)
C8	0.0394 (9)	0.0394 (9)	0.0362 (10)	−0.0009 (7)	0.0056 (7)	0.0017 (7)
C9	0.0423 (10)	0.0463 (10)	0.0463 (11)	0.0070 (8)	−0.0032 (8)	−0.0016 (9)
C10	0.0485 (10)	0.0402 (10)	0.0476 (12)	0.0084 (8)	0.0004 (9)	−0.0051 (8)
C11	0.0428 (9)	0.0380 (9)	0.0399 (10)	−0.0026 (7)	0.0038 (8)	0.0003 (8)
C12	0.0364 (9)	0.0493 (10)	0.0459 (11)	0.0039 (7)	0.0011 (8)	−0.0025 (9)
C13	0.0422 (10)	0.0439 (10)	0.0463 (11)	0.0056 (8)	0.0073 (8)	−0.0023 (8)
C14	0.0470 (10)	0.0422 (10)	0.0432 (11)	0.0000 (8)	0.0001 (8)	−0.0022 (8)
C15	0.0479 (10)	0.0413 (10)	0.0421 (11)	−0.0022 (8)	0.0051 (8)	−0.0011 (8)
C16	0.0563 (12)	0.0487 (11)	0.0455 (12)	−0.0081 (9)	0.0052 (9)	−0.0084 (9)
C17	0.0786 (15)	0.0489 (12)	0.0609 (15)	−0.0035 (11)	0.0173 (12)	−0.0134 (11)
N1	0.0489 (9)	0.0400 (8)	0.0430 (9)	0.0032 (7)	0.0056 (7)	0.0004 (7)
N2	0.0507 (10)	0.0511 (10)	0.0544 (11)	0.0084 (8)	−0.0106 (8)	−0.0151 (8)
O1	0.0578 (8)	0.0440 (7)	0.0478 (9)	0.0042 (6)	0.0004 (6)	−0.0084 (6)
O2	0.0596 (9)	0.0661 (10)	0.0640 (11)	0.0166 (7)	−0.0141 (8)	−0.0198 (8)
S1	0.0455 (3)	0.0623 (3)	0.0537 (4)	0.0105 (2)	−0.0047 (2)	−0.0168 (3)

### Geometric parameters (Å, °)

**Table:**

C1—C2	1.394 (3)	C10—H10	0.9300
C1—C6	1.398 (3)	C11—N2	1.367 (2)
C1—S1	1.731 (2)	C11—C12	1.407 (3)
C2—C3	1.374 (3)	C12—C13	1.370 (3)
C2—H2	0.9300	C12—H12	0.9300
C3—C4	1.392 (4)	C13—H13	0.9300
C3—H3	0.9300	C14—N2	1.434 (2)
C4—C5	1.367 (3)	C14—C15	1.507 (3)
C4—H4	0.9300	C14—H14A	0.9700
C5—C6	1.397 (3)	C14—H14B	0.9700
C5—H5	0.9300	C15—O2	1.197 (2)
C6—N1	1.385 (2)	C15—O1	1.318 (2)
C7—N1	1.300 (2)	C16—O1	1.456 (2)
C7—C8	1.460 (3)	C16—C17	1.488 (3)
C7—S1	1.7579 (18)	C16—H16A	0.9700
C8—C9	1.388 (3)	C16—H16B	0.9700
C8—C13	1.397 (3)	C17—H17A	0.9600
C9—C10	1.380 (3)	C17—H17B	0.9600
C9—H9	0.9300	C17—H17C	0.9600
C10—C11	1.395 (3)	N2—H2A	0.853 (9)
			
C2—C1—C6	122.01 (19)	C13—C12—C11	120.77 (17)
C2—C1—S1	128.85 (17)	C13—C12—H12	119.6
C6—C1—S1	109.13 (14)	C11—C12—H12	119.6
C3—C2—C1	117.9 (2)	C12—C13—C8	121.42 (17)
C3—C2—H2	121.1	C12—C13—H13	119.3
C1—C2—H2	121.1	C8—C13—H13	119.3
C2—C3—C4	120.7 (2)	N2—C14—C15	109.62 (15)
C2—C3—H3	119.6	N2—C14—H14A	109.7
C4—C3—H3	119.6	C15—C14—H14A	109.7
C5—C4—C3	121.4 (2)	N2—C14—H14B	109.7
C5—C4—H4	119.3	C15—C14—H14B	109.7
C3—C4—H4	119.3	H14A—C14—H14B	108.2
C4—C5—C6	119.4 (2)	O2—C15—O1	124.76 (18)
C4—C5—H5	120.3	O2—C15—C14	124.23 (18)
C6—C5—H5	120.3	O1—C15—C14	111.01 (16)
N1—C6—C5	125.91 (19)	O1—C16—C17	107.31 (17)
N1—C6—C1	115.52 (16)	O1—C16—H16A	110.3
C5—C6—C1	118.57 (19)	C17—C16—H16A	110.3
N1—C7—C8	124.46 (16)	O1—C16—H16B	110.3
N1—C7—S1	115.02 (14)	C17—C16—H16B	110.3
C8—C7—S1	120.52 (13)	H16A—C16—H16B	108.5
C9—C8—C13	117.59 (17)	C16—C17—H17A	109.5
C9—C8—C7	122.20 (16)	C16—C17—H17B	109.5
C13—C8—C7	120.21 (16)	H17A—C17—H17B	109.5
C10—C9—C8	121.81 (17)	C16—C17—H17C	109.5
C10—C9—H9	119.1	H17A—C17—H17C	109.5
C8—C9—H9	119.1	H17B—C17—H17C	109.5
C9—C10—C11	120.39 (17)	C7—N1—C6	110.97 (16)
C9—C10—H10	119.8	C11—N2—C14	123.52 (16)
C11—C10—H10	119.8	C11—N2—H2A	121.2 (16)
N2—C11—C10	122.80 (17)	C14—N2—H2A	114.6 (16)
N2—C11—C12	119.18 (16)	C15—O1—C16	116.58 (16)
C10—C11—C12	118.00 (17)	C1—S1—C7	89.35 (9)
			
C6—C1—C2—C3	−1.2 (3)	C10—C11—C12—C13	1.5 (3)
S1—C1—C2—C3	179.40 (17)	C11—C12—C13—C8	−0.1 (3)
C1—C2—C3—C4	0.8 (3)	C9—C8—C13—C12	−1.0 (3)
C2—C3—C4—C5	−0.1 (4)	C7—C8—C13—C12	178.72 (17)
C3—C4—C5—C6	−0.3 (3)	N2—C14—C15—O2	0.6 (3)
C4—C5—C6—N1	179.89 (19)	N2—C14—C15—O1	−179.36 (16)
C4—C5—C6—C1	0.0 (3)	C8—C7—N1—C6	180.00 (16)
C2—C1—C6—N1	−179.10 (18)	S1—C7—N1—C6	−0.2 (2)
S1—C1—C6—N1	0.4 (2)	C5—C6—N1—C7	179.97 (18)
C2—C1—C6—C5	0.8 (3)	C1—C6—N1—C7	−0.1 (2)
S1—C1—C6—C5	−179.68 (15)	C10—C11—N2—C14	8.1 (3)
N1—C7—C8—C9	179.13 (18)	C12—C11—N2—C14	−173.43 (19)
S1—C7—C8—C9	−0.6 (3)	C15—C14—N2—C11	177.26 (18)
N1—C7—C8—C13	−0.6 (3)	O2—C15—O1—C16	2.3 (3)
S1—C7—C8—C13	179.68 (14)	C14—C15—O1—C16	−177.66 (16)
C13—C8—C9—C10	0.7 (3)	C17—C16—O1—C15	176.34 (17)
C7—C8—C9—C10	−178.98 (18)	C2—C1—S1—C7	179.0 (2)
C8—C9—C10—C11	0.6 (3)	C6—C1—S1—C7	−0.41 (15)
C9—C10—C11—N2	176.82 (19)	N1—C7—S1—C1	0.39 (15)
C9—C10—C11—C12	−1.7 (3)	C8—C7—S1—C1	−179.83 (16)
N2—C11—C12—C13	−177.13 (19)		

### Hydrogen-bond geometry (Å, °)

**Table:**

*D*—H···*A*	*D*—H	H···*A*	*D*···*A*	*D*—H···*A*
C12—H12···O2^i^	0.93	2.60	3.390 (2)	144
N2—H2A···O2^i^	0.85 (1)	2.40 (1)	3.188 (2)	154 (2)

Symmetry codes: (i) −*x*−1, −*y*, −*z*+1.
